# Glutathione and the intracellular labile heme pool

**DOI:** 10.1007/s10534-020-00274-w

**Published:** 2020-12-10

**Authors:** Rosemary O’Keeffe, Gladys Oluyemisi Latunde-Dada, Yu-Lin Chen, Xiaole L. Kong, Agostino Cilibrizzi, Robert C. Hider

**Affiliations:** 1 Institute of Pharmaceutical Science, King’s College London, London, UK; 2 DMPK/ADME Research, Oncology R & D, Astrazenca, Cambridge, UK; 3 Department of Nutrition, King’s College London, London, UK

**Keywords:** Heme, Hematin, Glutathione

## Abstract

One candidate for the cytosolic labile iron pool is iron(II)glutathione. There is also a widely held opinion that an equivalent cytosolic labile heme pool exists and that this pool is important for the intracellular transfer of heme. Here we describe a study designed to characterise conjugates that form between heme and glutathione. In contrast to hydrated iron(II), heme reacts with glutathione, under aerobic conditions, to form the stable hematin–glutathione complex, which contains iron(III). Thus, glutathione is clearly not the cytosolic ligand for heme, indeed we demonstrate that the rate of heme degradation is enhanced in the presence of glutathione. We suggest that the concentration of heme in the cytosol is extremely low and that intracellular heme transfer occurs via intracellular membrane structures. Should any heme inadvertently escape into the cytosol, it would be rapidly conjugated to glutathione thereby protecting the cell from the toxic effects of heme.

## Introduction

The labile iron pool, first proposed by Greenberg and Wintrope (1946) plays an essential role in supplying iron to iron–dependent enzymes and to mitochondria for heme and iron–sulfur cluster synthesis (Hider and Kong 2011). On the basis of a range of biochemical and thermodynamic arguments, Williams (1982) suggested that the oxidation state of cytosolic iron is iron(II) and that its concentration falls in the range 10^−7^–10^−6^ M, a proposal supported by fluorescence studies (Breuer et al 1995). Evidence has been presented for the nature of this labile iron pool as being iron(II)glutathione (Hider and Kong 2011), GSH acting as a buffer for iron(II), maintaining iron in the reduced state. An iron chaperone (PCBP–1) has been demonstrated to only donate iron for cytosolic [2Fe–2S] cluster assembly as a PCBP–1–Fe–GSH complex, no transfer of iron occurring in the absence of GSH. PCBP–1 apparently requires GSH to bind iron (Patel et al 2019).

A labile heme cytosolic pool is also reported to exist in mammalian cells (Shviro and Shaklai 1987; Sahini et al 1996) and as the axial iron coordination sites of heme are unoccupied, it is conceivable that GSH coordinates iron at one of these sites. Directly relating to this suggestion is the finding of Shviro and Shaklai (1987) that hematin binds GSH and that the 1:1 species is the dominant form of hematin (hematin is [iron^III^(OH) protoporphyrin IX], hemin is [iron^III^(Cl) protoporphyrin IX]) in the cytosol of erythrocytes. This finding was subsequently confirmed by Sahini et al (1996) who also demonstrated that GSH binds to hematin. These latter workers suggested that “GSH may serve physiologically as the cytosolic ligand of free hematin”. In view of these observations we decided to further characterise the interaction between GSH and heme.

## Materials and methods

### Chemicals

Reduced glutathione was purchased from Santa Cruz Biotechnology, L–ascorbic acid from Acros Organics, other chemicals were from Sigma. Solvents were from Fisher Scientific.

### *Spectroscopy of hematin*–*containing solutions*

Hematin (50 µM) and glutathione (0–5 mM) were dissolved in potassium phosphate buffer (10 mM, pH 8.0) containing potassium chloride (20 mM). The absorbance of the hematin solutions was measured at 1 nm intervals between 550 nm and 750 nm using a PerkinElmer UV/Vis spectrophotometer Lambda 2 coupled with Lambda25 software. The absorption difference between 618 nm and 655 nm was plotted against the log concentration of glutathione. The affinity constant between GSH and hematin was determined from this plot. The HySS2009 programme (Alderighi et al 1999), was used to provide the corresponding speciation plots.

### Mass spectrometry

A solution of hematin (1.3 mg, 1 equiv) in dimethyl formamide (1 mL) was mixed with a solution of glutathione (6.1 mg, 10 equiv) in phosphate buffer 1 × containing 20 mM of potassium chloride (pH = 7.5). The mixture was sonicated for 20 min (recorded pH = 7.2). After 24 h, the solution was separated from a small amount of precipitate formed. Samples for mass spectrometry were prepared by diluting an aliquot of the solution with 50:50 acetonitrile/water + 0.1% formic acid, or by adding 2–3 drops of DMSO to the precipitate before dilution with 50:50 acetonitrile/water + 0.1% formic acid. Samples were directly infused into a Waters Micromass ZQ mass spectrometer, using a 250 µL syringe. Low–resolution mass spectrometry analyses were performed in positive (ES^+^) and negative (ES^−^) ion mode and data were processed with MassLynx software (Waters).

### Hematin stability in the presence of hydrogen peroxide

The absorbance of the hematin and GS–hematin in solution was measured between 575 nm and 700 nm at 1 nm intervals using PerkinElmer UV/Vis spectrophotometer Lambda 2 coupled with Lambda25 software. H_2_O_2_ (1 mL) was added to the solutions to give a final concentration of 0–25 µM and final concentrations of 10 µM hematin and 0–2 mM glutathione. The average change in absorbance at 618 nm for hematin solutions, and 655 nm for GS–hematin solutions were plotted against time.

### *Incubation of hematin and GS*–*hematin with liposomes*

Phosphatidylcholine from egg yolk (200 mg) was dissolved in 40 mL chloroform in a 250 mL round bottom flask. The mixture was reduced to dryness by rotary evaporation. KCl (150 mM, 40 mL) buffered with potassium phosphate (pH 8.0, 10 mM) was added. Liposomes were incubated overnight at room temperature with shaking (25 rpm). The resulting multilamellar liposomes were centrifuged at 3000 rpm for 5 min. The supernatant was aspirated and the liposomes were resuspended in KCl (150 mM) buffered with phosphate (pH 8.0, 10 mM). The liposomes were washed twice and finally suspended in phosphate buffered KCl (150 mM, 8 mL).

Phosphatidylcholine and cholesterol were dissolved in chloroform (40 mL) in a 1:1 molar ratio (125.2 mg phosphatidylcholine, 74.8 mg cholesterol) and treated as above. Phosphatidylcholine, cholesterol and dicetyl phosphate were dissolved in a mix of chloroform (40 mL) and ethanol (20 mL) in a 2:2:1 molar ratio (59.2 mg phosphatidylcholine, 59.2 mg cholesterol, 41.8 mg dicetyl phosphate) and processed as above.

Hematin (10 µM) was dissolved in KCl (150 mM) buffered to pH 8.0 with 10 mM phosphate, in the presence or absence of GSH (2 mM). The mixture was vortexed for 10 min, centrifuged at 4000 rpm for 5 min and the supernatant decanted. Triplicate solutions (20 mL) of hematin ± glutathione were added to phosphatidylcholine, phosphatidylcholine–cholesterol, and phosphatidylcholine–cholesterol–dicetyl phosphate liposomes, and incubated at 37 °C with shaking (225 rpm). Aliquots (4 mL) were taken after 15 min, 1 h, 2 h, and 4 h. The aliquots were centrifuged at 4000 rpm for 10 min, and the supernatant discarded. The pellets were dissolved in DMSO (1.5 mL) by shaking (70 rpm) overnight. The resulting solution was centrifuged at 4000 rpm for 10 min and the absorbance of the supernatant was recorded at 416 nm.

### *Hematin and GS*–*hematin stability in the presence of ascorbic acid*

Hematin was dissolved in KCl (20 mM) buffered with potassium phosphate (10 mM, pH 8.0) with or without glutathione, to a final concentration of either 10 µM or 1 µM. Solutions were vortexed for 10 min, centrifuged at 4000 rpm for 10 min and supernatant transferred to quartz cuvette. Ascorbic acid (1 mL) was added to a final concentration of 300 µM. The absorbance at 550 nm was monitored with time.

For conditions under either 5% oxygen or 100% nitrogen the solutions were stirred under the relevant gas for 30 min whereupon the cuvette was sealed.

## Results

### Interaction of glutathione with hematin

**Fig. 1:**
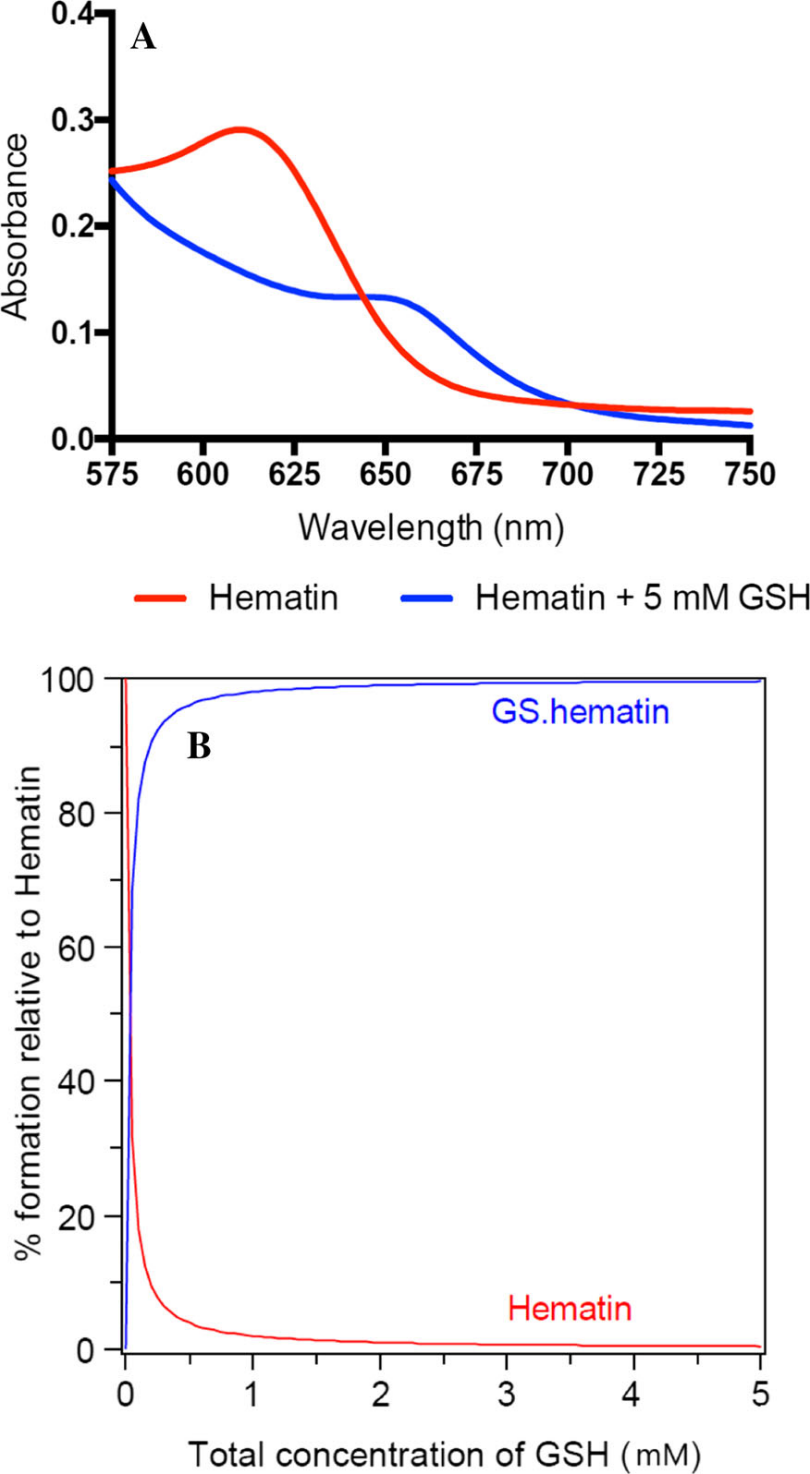
Absorption spectrum and speciation plot of hematin ± glutathione. (**a**) Hematin was dissolved to a final concentration of 10 µM in 10 mM phosphate buffer, 20 mM KCl, pH 8.0 (red line) or 10 mM phosphate buffer, 20 mM KCl, 5 mM glutathione, pH 8.0 (blue line). (**b**) Speciation plot of GS·hematin [hematin]_total_ = 10 µM; [GSH] = 0–5 mM

**Fig. 2:**
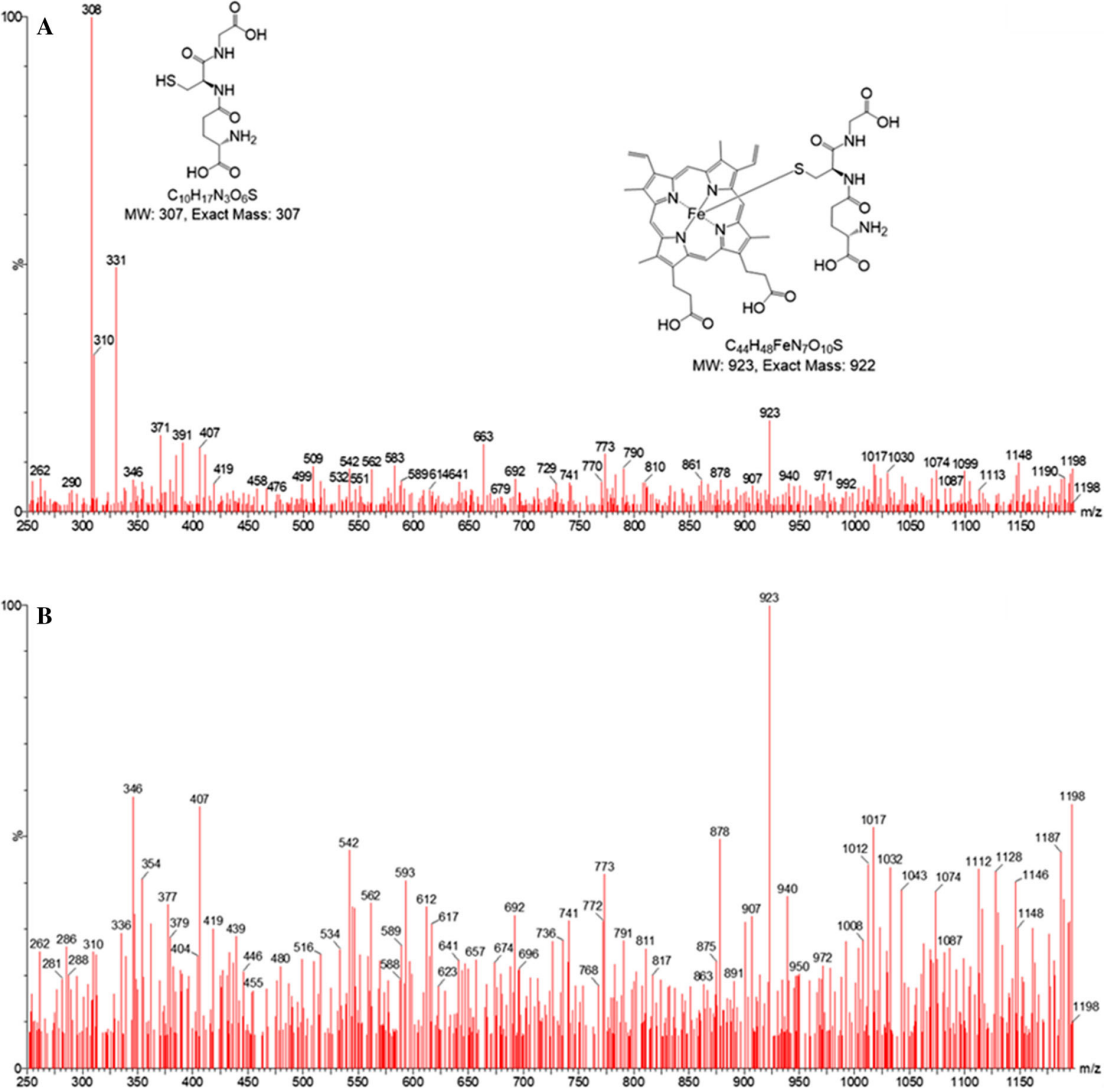
LR–MS of hematin–glutathione samples prepared from (**a**) the reaction solution and (**b**) the precipitate. Spectra have been acquired in positive ion mode (ES^+^), in the range 250–1200 m*/z*. The net charge of the 923 species arises from: + 3 (Fe III), −2 (protoporphyrin IX), −1 (GSH), and + 1 (proton). If the redox state of iron was iron (II), the 923 species would be neutral and would not provide a mass peak under ES^+^ conditions. In order to provide such a peak, the Fe^2+^-complex would require the addition of 2 protons, which would no longer correspond to a 923 species

When glutathione (5 mM) was mixed with hematin (20 µM) in phosphate buffer (10 mM, pH 8.0) containing potassium chloride (20 mM), a shift in the peak absorbance from 618 to 655 nm was observed (Fig. 1a). This is in agreement with previous studies (Shviro and Shaklai 1987). It is clear that, in contrast to iron(II)glutathione, in the presence of a porphyrin ring the favoured redox state is iron(III). This redox state was confirmed by mass spectrometry, where in the presence of glutathione, a new peak appeared at 923 m*/z* which corresponds to the [M + H]^+^ of GS/hematin 1:1 conjugate (Fig. 2). This peak is present in the samples prepared from both the reaction solution (Fig. 2a) and the precipitate (Fig. 2b). The peak at 308 m*/z* (Fig. 2a) in the analysis of the reaction solution corresponds to glutathione, i.e. [M + H]^+^. A peak at 308 m*/z* is not detected in the analysis of the sample prepared from the precipitate (Fig. 2b), where the peak at 923 m*/z* (corresponding to GS/hematin conjugate) has the highest intensity. The 923 m/z peak corresponds to an iron(III) complex and not an iron(II) complex (see legend of Fig. 2). This finding is in agreement with observations made by previous workers (Shviro and Shaklai 1987; Sahini et al. 1996). Hematin (20 µM) was dissolved in varying concentrations of GSH (0–5 mM) and the difference in absorption between 618 nm and 655 nm was plotted against GSH concentration, leading to the determination of the affinity constant, Ka, 5.0 × 10^4^ M^−1^. The resulting speciation plot indicated that 99% of hematin was coordinated by GSH under physiological conditions. (Fig. 1b).

### *GS*–*hematin stability in the presence of hydrogen peroxide*

**Fig. 3:**
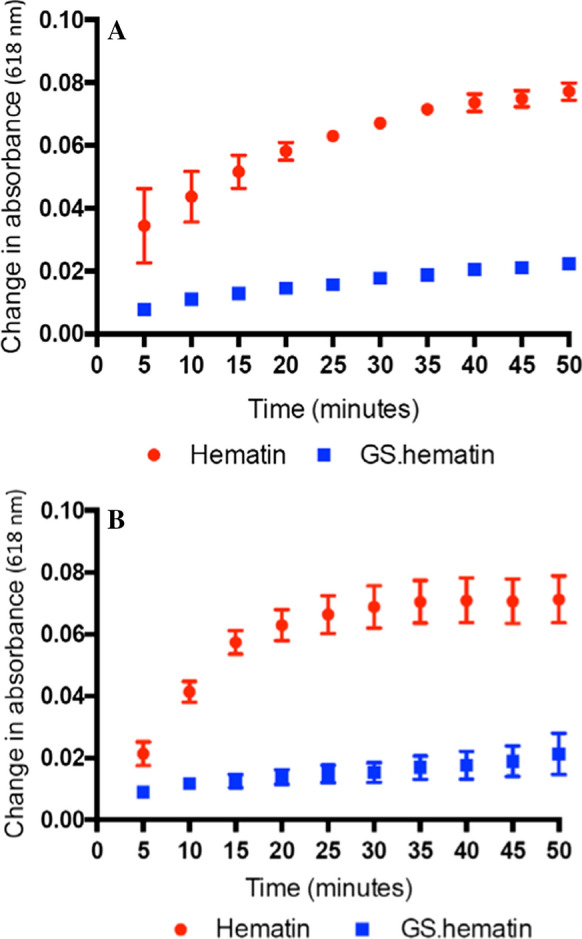
Degradation of hematin and GS.hematin in the presence of H_2_O_2_ over time. (**a**), 5 µM H_2_O_2_, (**b**), 10 µM H_2_O_2_. Hematin (10 µM) (Hematin, red cirlce) or GS.hematin (GS.hematin, blue squares) (10 µM hematin, 2 mM glutathione) were dissolved in 10 mM phosphate buffer, 20 mM KCl, pH 8.0

The degradation of hematin in the presence and absence of GSH (2 mM) induced by H_2_O_2_ was monitored by the change of absorption at 618 nm. Whereas there was a major change in absorption with hematin alone, there was only a minor change observed in the presence of GSH (Fig. 3), at both 5 µM and 10 µM H_2_O_2_. Clearly GSH protects hematin from oxidative damage by H_2_O_2_.

### *GS*–*hematin partitioning into liposomes*

**Fig. 4:**
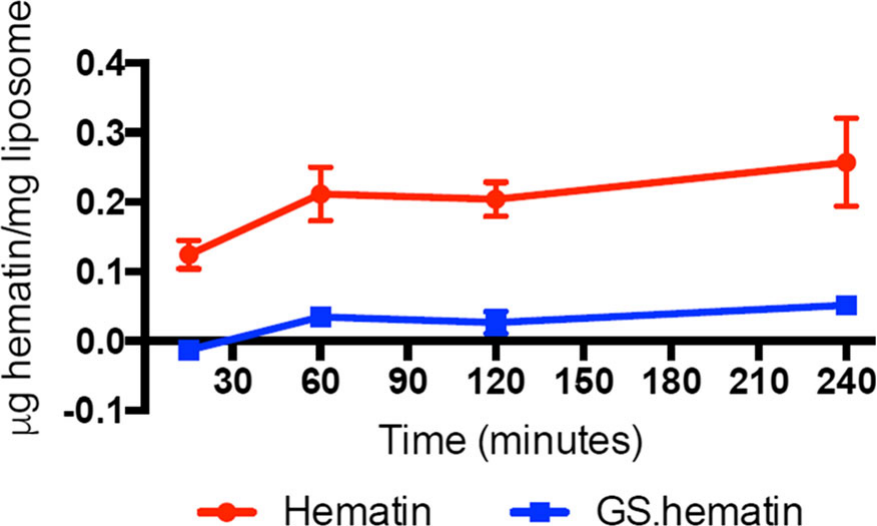
Effect of glutathione on hematin partitioning into phosphatidylcholine liposomes. Hematin (10 µM) dissolved in either; 10 mM phosphate buffer, 150 mM KCl, pH 8.0 (Hematin, red cirlce) or 10 mM phosphate buffer, 150 mM KCl, 2 mM glutathione, pH 8.0 (GS.hematin, blue squares) and incubated at 37 °C with phosphatidylcholine liposomes

**Fig. 5:**
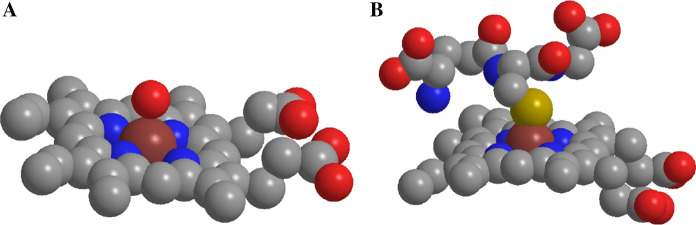
Structure of hematin and GS.hematin. Carbon is depicted in grey, oxygen in red, nitrogen in blue, iron in brown and sulfur in yellow. (**a**)—Hematin structure, the two hydrophilic carboxylate groups of the porphyrin ring are positioned at one end of the ring whilst the hydrophobic vinyl groups are at the opposite end of the planar molecule. (**b**)—GS.hematin structure, with the addition of two carboxylate groups and an amine group the complex is less hydrophobic and more bulky when compared to the planar hematin molecule

Hematin is known to rapidly partition into lipid bilayers, where the iron can redox cycle, resulting in lipid peroxidation (Vincent 1989; Kumar and Bandyopadhyay 2005). In order to establish whether GSH interferes with the partitioning of hematin, the GS–hematin complex was incubated with phosphatidylcholine liposomes. In contrast to hematin, which partitions rapidly (within 15 min), GS–hematin was found to partition into the liposome bilayers at a much slower rate (Fig. 4). Similar effects were observed with cholesterol- and dicetyl phosphate–containing phosphatidyl choline liposomes. Complexation of hematin with GSH creates a bulkier hydrophilic molecule, which contrasts markedly with the amphiphilic planar structure of hematin (Fig. 5) and this offers a ready explanation for the differential partitioning properties of these two molecules.

### *GS*–*hematin stability in the presence of ascorbic acid*

**Fig. 6:**
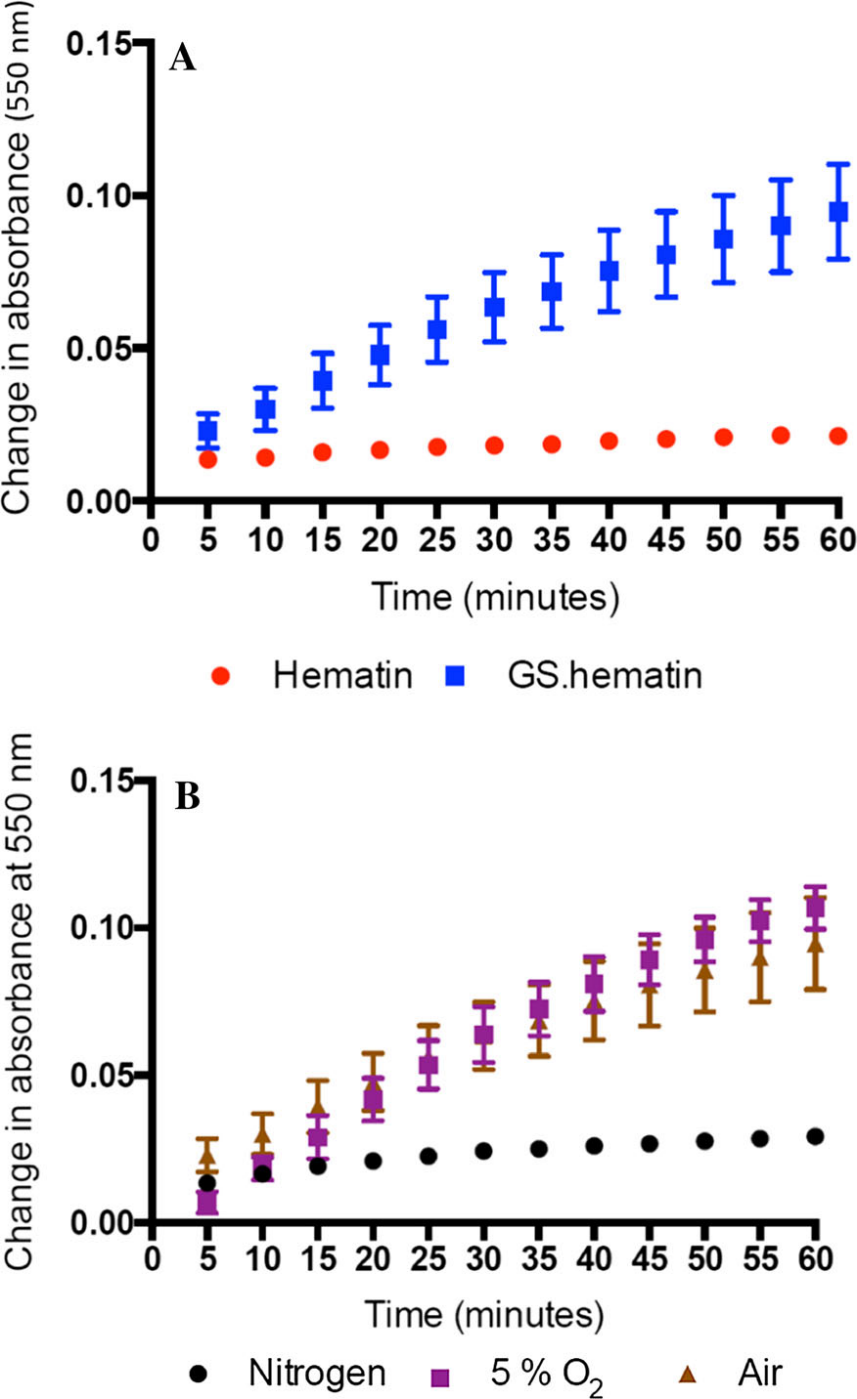
Degradation of hematin and GS.hematin in the presence of ascorbic acid. (**a**) Hematin (10 µM) (Hematin, red cirlce) or GS.hematin (GS.hematin, blue squares) (10 µM hematin, 2 mM glutathione) was dissolved in 10 mM phosphate buffer, 20 mM KCl, pH 8.0 and absorbance recorded at 550 nm (GS.hematin) and 618 nm (hematin). Ascorbic acid (300 µM) was added to the solutions. (**b**) Hematin (10 µM) was dissolved in 10 mM phosphate buffer, 20 mM KCl, 2 mM glutathione, pH 8.0 and either left for 30 min (air, gold triangles), had 100% N_2_ (nitrogen, black circles) or 90% N_2_, 5% CO_2_, 5% O_2_ (5% O_2_, purple squares) bubbled through the solution for 30 min. Ascorbic acid (300 µM) was added to the solutions

When hematin was incubated with ascorbic acid (300 µM) for 1 h there was virtually no change in the visible spectrum. This contrasts with the behaviour of GS–hematin when treated in an identical fashion. In the latter case the peaks at 550 nm and 655 nm were decreased. If ascorbic acid caused glutathione to dissociate from hematin, the spectrum of either hematin or heme should appear, but this was not the case. Thus, the ascorbic acid- induced spectral change is likely to result from oxidation and breakdown of the porphyrin ring. The changes in absorbance at 550 nm are presented in Fig. 6a. The process is dependent on the presence of oxygen as no spectral changes were observed under nitrogen (Fig. 6b). It is likely that a redox reaction between reduced ascorbic acid and hematin generates free radicals, which in turn leads to the degradation of the porphyrin ring.

## Discussion

This investigation was initiated on the premise that reduced glutathione could form a 1:1 adduct with heme in an analogous fashion to the adduct formation with inorganic iron(II), namely iron(II)glutathione (Hider and Kong 2011). Should it form, it would be a strong candidate for the cytosolic labile heme pool. However it emerged, as previously reported by others (Shviro and Shaklai 1987; Sahini et al 1996), that although heme reacts rapidly with glutathione, the product is the 1:1 adduct of glutathione and hematin which holds iron in the iron(III) oxidation state. This renders it unlikely for glutathione to act as the heme ligand in the cytosolic heme pool. Significantly glutathione renders hematin more susceptible towards oxidative degradation in the presence of physiological levels of ascorbic acid (Fig. 6). This finding again indicates that the GSH/hematin complex does not function as the endogenous cytosolic protoporphyrin–iron pool.

Heme is lipophilic and intercalates readily into membranes; it has an intrinsic peroxidase activity and can generate reactive oxygen species by redox cycling. It is thus essential that intracellular heme is not allowed to exist in an unbound state and must either be bound to a chaperone molecule or be enclosed in intracellular membrane structures, for instance the endoplasmic reticulum. Heme uptake by enterocytes has been reported to be mediated by a receptor—mediated endocytic pathway (Gräsbeck et al 1979; Worthington et al 2001), the absorbed heme, which rapidly enters lysosomes (Wyllie and Kaufman 1982), is probably not exposed to the cytoplasm. Newly synthesised heme may also not be exposed to the cytoplasm on leaving the mitochondria, instead achieving transfer either by inter-organellar transport via vesicles or by heme transfer during direct contact between inter-organellar membranes (Severance and Hamza 2009). In mammals the bulk of heme breakdown is achieved during the processing of aged erythrocytes by macrophages. This process of erythrophagocytosis leads to the breakdown of hemoglobin and the oxidation of heme in macrophage lysosomes. The resulting iron being released into the cytoplasm as hydrated iron(II) via the DMT–1 transporter. Thus, at each of these three stages of heme transfer, namely cell uptake, release from mitochondria and macrophage breakdown there is no necessity for heme to enter the cytosol. The transfer of heme via an intermediate compartment would minimise the release of cytotoxic heme into the cytoplasm and facilitate the regulated movement of heme (Severance and Hamza 2009). Indeed, in view of the toxic nature of free heme it seems likely that its concentration in the cytoplasm should be vanishing small. However, if heme should inadvertently enter the cytoplasm; it would be rapidly bound by glutathione and oxidised to GS–hematin thus preventing the iron from redox cycling in the presence of H_2_O_2_ (Fig. 3) and minimising the partitioning of hematin into membranes (Fig. 4). Furthermore, in the presence of Vitamin C it would be degraded probably in a manner similar to that of heme oxygenase, the resulting breakdown products being digested in lysosomes (Fig. 6). These three properties will act cooperatively so minimising the toxicity of any released heme into the cytoplasm.

## Conclusion

Although glutathione is able to coordinate heme bound iron, forming GS–hematin, the affinity of glutathione for hematin is such that any heme or hematin generated in the cytoplasm will be almost totally bound to glutathione, forming a 1:1 complex. Thus unlike the cytosolic labile iron pool where the redox state of iron is buffered in the iron(II) state by glutathione, in aerobic cells cytosolic heme will be rapidly converted to hematin in the presence of physiological levels of glutathione. It is suggested that glutathione acts to remove any heme that has been inadvertently released into the cytoplasm, and that in normal circumstances there will be extremely low levels of heme in the cytoplasm, intracellular transfer of heme being conducted by vesicles and/or direct contact between intracellular membranes.
